# Large-scale gene expression analysis reveals robust gene signatures for prognosis prediction in lung adenocarcinoma

**DOI:** 10.7717/peerj.6980

**Published:** 2019-06-03

**Authors:** Yiyan Songyang, Wei Zhu, Cong Liu, Lin-lin Li, Wei Hu, Qun Zhou, Han Zhang, Wen Li, Dejia Li

**Affiliations:** 1 Department of Occupational and Environmental Health, School of Public Health, Wuhan University, Wuhan, China; 2 Department of Emergency, Renmin Hospital of Wuhan University, Wuhan, China

**Keywords:** Large-scale gene expression analysis, Hyperactive G2-M transition, MMPC algorithm, Overall survival, Lung adenocarcinoma (LUAD)

## Abstract

Lung adenocarcinoma (LUAD) is the leading cause of cancer-related death worldwide. High mortality in LUAD motivates us to stratify the patients into high- and low-risk groups, which is beneficial for the clinicians to design a personalized therapeutic regimen. To robustly predict the risk, we identified a set of robust prognostic gene signatures and critical pathways based on ten gene expression datasets by the meta-analysis-based Cox regression model, 25 of which were selected as predictors of multivariable Cox regression model by MMPC algorithm. Gene set enrichment analysis (GSEA) identified the Aurora-A pathway, the Aurora-B pathway, and the FOXM1 transcription factor network as prognostic pathways in LUAD. Moreover, the three prognostic pathways were also the biological processes of G2-M transition, suggesting that hyperactive G2-M transition in cell cycle was an indicator of poor prognosis in LUAD. The validation in the independent datasets suggested that overall survival differences were observed not only in all LUAD patients, but also in those with a specific TNM stage, gender, and age group. The comprehensive analysis demonstrated that prognostic signatures and the prognostic model by the large-scale gene expression analysis were more robust than models built by single data based gene signatures in LUAD overall survival prediction.

## Introduction

Lung adenocarcinoma (LUAD) is the leading cause of cancer-related death worldwide (Siegel, Miller & Jemal, 2015). Risk factors include smoking, age, family history, air pollution, etc. (Malhotra et al., 2016). The lung adenocarcinoma is most commonly diagnosed at a late stage, which results in a poor patient survival rate (Salomaa et al., 2005). Current therapies incorporate surgical, medical, and radio-therapeutic interventions. However, the long-term survival rate of patients diagnosed with primary LUAD has not been improved (Field & Raji, 2010).

The prognosis of lung cancer mainly depends on the probability of recurrence and metastasis (Yang, 2009). Although the TNM staging system had the potential to predict the prognosis, its performance was still not satisfactory (Marchevsky, 2006). Recently, many efforts were made to identify the potential molecules that are the prognostic markers of lung cancer patients (Chen et al., 2018; Li et al., 2017; Park et al., 2012; Shukla et al., 2017). With the advances in microarray and RNA sequencing technologies, gene expression signatures were widely applied to predicting the prognosis of lung adenocarcinoma. For example, Dama et al. reported a 10-gene signature able to predict prognosis of patients with stage I lung adenocarcinoma (Dama et al., 2017), which distinguishes an aggressive subtype from the early-stage LUAD. Wan et al. (2010) identified a 12-gene signature for lung cancer prognosis and chemo-response prediction. Moreover, Xu et al. identified a five-gene and corresponding protein signature for stage-I lung adenocarcinoma prognosis (Kadara et al., 2011). However, the gene signatures used for prognostic prediction by different studies are diverse from each other due to different methodologies, experimental platforms, batch effect, and other factors, which motivates us that a set of robust prognostic gene signatures are urgently needed for clinical study and application.

In the present study, we collected ten gene expression datasets of lung cancer from Gene Expression Omnibus (GEO) or ArrayExpress databases, which comprised 1,308 adenocarcinoma and 903 other etiologies. The meta-analysis-based Cox regression analysis identified a set of robust gene signatures and critical pathways associated with LUAD overall survival. Moreover, we also employed MMPC algorithm, which stands for Max-Min Parents and Children, to select gene signatures for multivariable Cox regression model. The multivariable Cox regression model not only exhibited robust performance in the training and validation sets, but also had the capability of predicting LUAD prognosis within TNM stages. The present study not only provided a set of robust gene signatures for prognosis prediction, but also facilitated our understanding of the mechanism of LUAD progression.

## Materials & Methods

### Data collection and pre-processing

Gene expression datasets were obtained from the NCBI Gene Expression Omnibus (GEO) (http://www.ncbi.nlm.nih.gov/geo) and ArrayExpress (http://www.ebi.ac.uk/arrayexpress/) databases. Prior to downstream analysis, we firstly mapped the array probes to the respective gene symbol by using the array annotations. To calculate the gene expression more conveniently, we used the average expression values of genes matching multiple probes.

### Binarization of gene expression levels from multiple datasets

The first seven datasets used in this study was merged by Lim’s merging method to remove batch effect as they were produced by the same microarray platform (Table 1). For each gene of the merged dataset and the 3 additional datasets, the expression values were binarized as high or low expression when the expression values higher or lower than its corresponding median, respectively. Based on the binarized gene expression pattern for each gene and each sample, we then merged the seven datasets and three addition datasets.

**Table 1:** Sample size and number of deceased patients for the ten lung adenocarcinoma gene expression datasets.

Datasets	# of patients	# of deceased patients	Stage (percent of stage I and II)	Age			Gender (percent of male)	Smoking
				5% quantile	median	95% quantile		
GSE10245	14	7	NA	NA	NA	NA	NA	NA
GSE10445	21	13	85.71%	48	53	56	74.19% (*n* = 21)	74.47% (*n* = 17)
GSE19188	87	64	100%	NA	NA	NA	74.6% (*n* = 81)	55.17% (*n* = 87)
GSE28571	80	80	100%	NA	NA	NA	NA	42.5% (*n* = 80)
GSE31210	57	33	98.25%	49	52	55	40.35% (*n* = 57)	82.69% (*n* = 52)
GSE33356	18	10	94.44%	47.25	51.5	55	0% (*n* = 18)	61.54% (*n* = 13)
GSE50081	32	16	46.88%	51.635	73.125	74.515	46.88% (*n* = 32)	85.71% (*n* = 14)
GSE68465	443	236	NA	58	64	72	50.34% (*n* = 443)	85.96% (*n* = 349)
GSE67639	439	233	84.21%	54	63	67	49.58% (*n* = 439)	NA
GSE13213	117	49	80.34%	55	61	67	51.28% (*n* = 117)	NA

### Overrepresentation enrichment analysis (ORA)

Overrepresentation enrichment analysis, which used hypergeometric test, was also implemented at WEB-based Gene Set Analysis Toolkit (WebGestalt) (Wang et al., 2017). The Reactome pathways were selected as the functional database (Fabregat et al., 2018). We chose 0.05 as the threshold of the *p*-value for significant pathways.

### Gene set enrichment analysis

The gene set enrichment analysis was implemented in javaGSEA (Subramanian et al., 2005) (version 3.0). The database with GMT files was customized by NCI-PID pathways (Schaefer et al., 2009) selected from all canonical pathways. The genes were pre-ranked based on the Z statistic in Cox model. 10,000 permutations were used to calculate the enrichment significance.

### Cox-regression based survival analysis

Cox-regression model was used to evaluate the differences of overall survival between patients from two conditions. This analysis was implemented in R programming software (R Core Team, 2018) with the *survdiff* function. To visualize the overall survival for each group, we used Kaplan–Meier curves to estimate the survival probability. The *hazard.ratio* function in *survcomp* package (Haibe-Kains et al., 2008) was used to calculate the hazard ratios and corresponding *p*-values. The risk score for each patient was predicted by the Cox model with “linear predictor” type based on the 25 genes selected by MMPC algorithm (Brown, Tsamardinos & Aliferis, 2004), which was implemented in predict.coxph function.

## Results

### Summary of enrolled datasets for discovery

A total of 10 non-small cell lung cancer (NSCLC) gene expression datasets were collected from Gene Expression Omnibus (GEO) or ArrayExpress database. Tumor samples should be characterized by primary lung adenocarcinoma histology, and with overall survival. Notably, 309 tumor samples from seven datasets, including GSE10245 (Kuner et al., 2009), GSE10445 (Broet et al., 2009), GSE19188 (Hou et al., 2010), GSE28571 (Micke et al., 2011), GSE31210 (Okayama et al., 2012), GSE33356 (Lu et al., 2011), and GSE50081 (Der et al., 2014), were produced by the same microarray platform (Affymetrix Human Genome U133 Plus 2.0 Array), which were merged and normalized by Lim et al. (2018). In addition, another three datasets, GSE68465 (Director’s Challenge Consortium for the Molecular Classification of Lung et al., 2008), GSE67639 (Roepman et al., 2009), and GSE13213 (Tomida et al., 2009), were also incorporated in the present study. Finally, a total of 1,308 LUAD cases were collected for further analysis, 741 (56.65%) of whom were dead (Table 1).

### Identification of prognostic genes by meta-analysis-based Cox regression model

**Figure 1:**
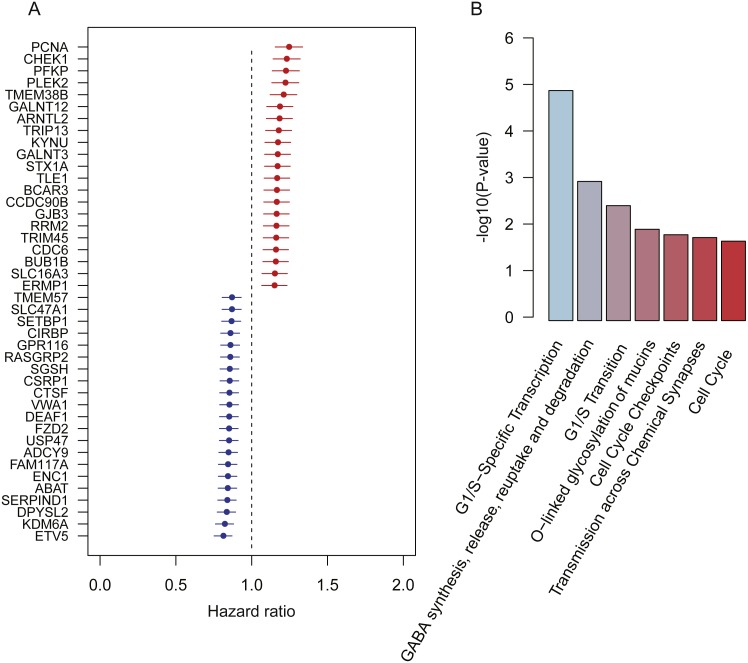
Prognostic genes identified by meta-analysis-based Cox regression analysis. (A) 42 prognostic genes are ordered by hazard ratio. (B) The significance (−log_10_ (*p*-value)) of seven pathways enriched by the 42 prognostic genes are represented by the bars.

To robustly identify the prognostic genes associated with overall survival of lung adenocarcinoma, we integrated the ten gene expression datasets, and discretized the normalized expression value for each gene as high and low expression status within each dataset, which could avoid the batch effect by different platforms. Cox proportional hazard regression analysis was then performed on the discretized expression status for each gene. Given a stringent threshold at BH-adjusted *p*-value < 0.01, we successfully identified 42 genes significantly associated with LUAD overall survival, including 21 positively and 21 reversely correlated genes (Fig. 1A).

To further investigate functional roles of the prognostic genes, we performed overrepresentation enrichment analysis (ORA) on these genes. We identified seven pathways significantly enriched by the prognostic genes (Fig. 1B, *p*-value < 0.05). Remarkably, the cell cycle genes, such as *CHEK1*, *PCNA*, *RRM2*, *BUB1B*, and *CDC6*, were reversely correlated with patients’ overall survival, which were significantly enriched in pathways, such as G1/S-specific transcription, G1/S transition, cell cycle checkpoints, and cell cycle. Moreover, *GALNT3* and *GALNT12*, also reversely correlated with overall survival, were involved in O-linked glycosylation of mucins, indicating that O-linked glycosylation of mucins played key roles in LUAD progression. In addition, we also identified two prognostic genes, *ABAT* and *STX1A*, which participated in GABA synthesis, release, reuptake and degradation. Notably, cell cycle (Sithanandam et al., 2003), O-linked glycosylation of mucins (Hauselmann & Borsig, 2014), and GABA synthesis, release, reuptake, and degradation (Al-Wadei et al., 2012) have been reported to be involved in tumorigenesis or tumor progression. The results based on the enrichment analysis indicated that pathways such as cell cycle, O-linked glycosylation of mucins, and GABA synthesis, release, reuptake and degradation were the hallmarks of tumor progression and short overall survival.

### Identification of prognostic pathways by GSEA

**Figure 2:**
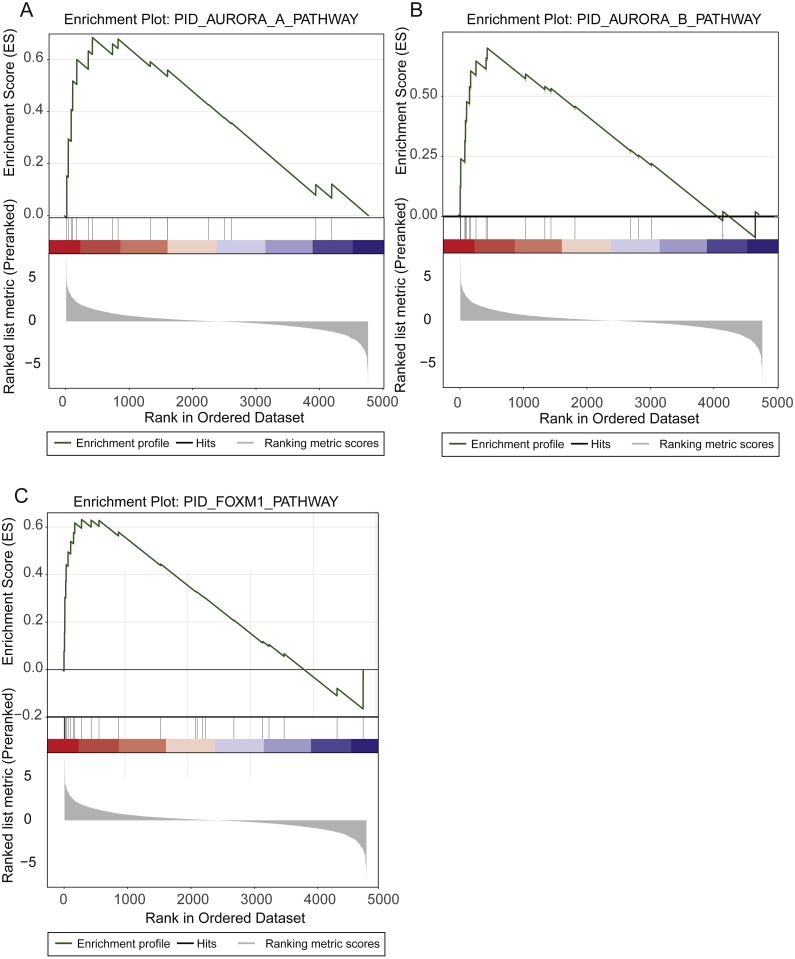
Prognostic pathways identified by gene set enrichment analysis (GSEA). The enrichment plots for Aurora-A signaling, Aurora-B signaling, and FOXM1 transcription network were illustrated in (A), (B), and (C).

To identify the prognostic pathways for LUAD patients, we ranked the genes based on their significance levels by Cox regression-based meta-analysis. The gene set enrichment analysis was then performed on the ranked gene set. Given the stringent thresholds (FDR < 0.05 for pathways, and log-rank test *p*-value < 0.05 for pathway genes in core enrichment), we identified the Aurora-A pathway, the Aurora-B pathway, and the FOXM1 transcription factor network as prognostic pathways in LUAD (Fig. 2).

The Aurora-A and Aurora-B pathways were responsible for G2-M transition in cell cycle (Sithanandam et al., 2003), and the expression levels of two key kinases, Aurora-A and Aurora-B, were significantly higher in high-risk group than low-risk group (Figs. 2A–2C). As FOXM1 is a transcription factor, which was a famous oncogene (Gartel, 2017), its target genes, such as *CCNA2*, *CCNB1*, *CCNB2*, *CCNE1*, *TGFA*, *BIRC5*, *CDK2*, *CENPF*, *CENPA*, and *AURKB*, were closely associated with overall survival of LUAD patients. Particularly, the transcription factor FOXM1, overexpression of which could significantly shorten the overall survival of LUAD patients, was also involved in G2-M transition (Fig. 2C). The result suggested that hyperactive G2-M transition in cell cycle was an indicator of poor prognosis in LUAD.

### Development of a gene expression signature-based prognostic model in LUAD

**Figure 3:**
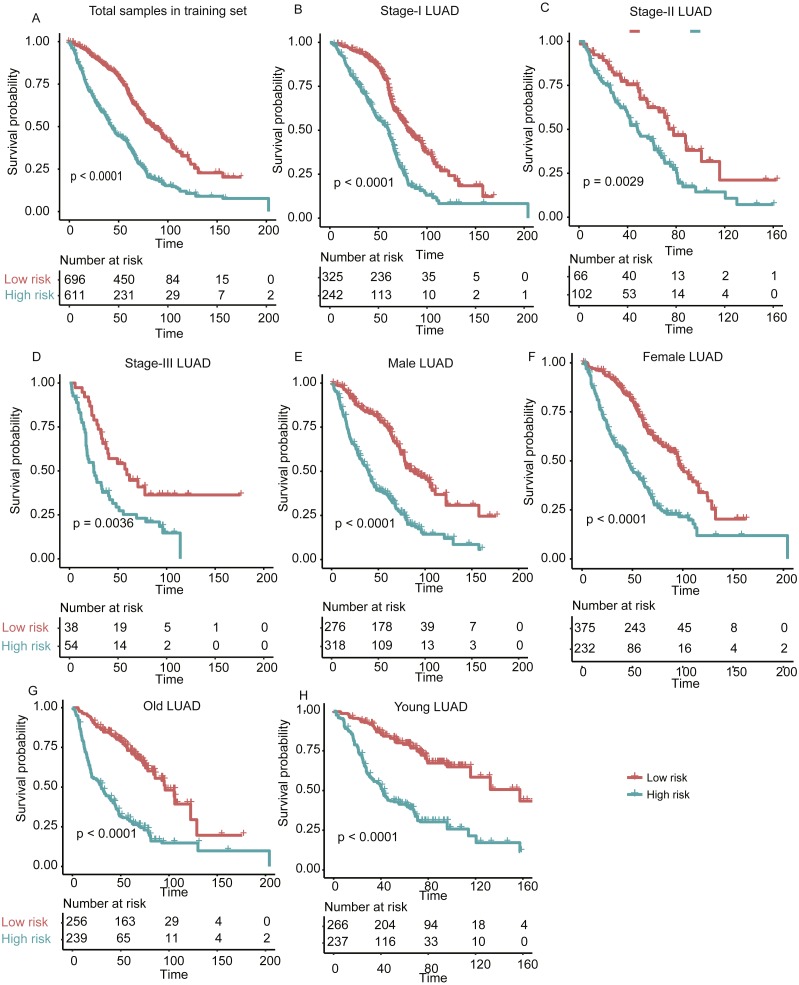
The performance of the stratification for the lung adenocarcinoma in training set based on the prognostic model. (A) The Kaplan–Meier curves of the poor and good prognosis groups show significant overall survival difference. (B, C, and D) showed the prognostic significance of the stratification in specific TNM stage, and (E and F) and (G and H) showed the survival difference between patients of high- and low-risk group from specific gender and age group, respectively.

As we described above, the univariate Cox proportional hazard regression analysis successfully identified 42 prognostic genes. To further select signatures used for multivariable Cox regression model, we employed MMPC algorithm, which is a constraint based feature selection algorithm (Brown, Tsamardinos & Aliferis, 2004). We then selected 25 genes from the 42 prognostic genes, including *ABAT*, *BCAR3*, *CTSF*, *DEAF1*, *ENC1*, *ETV5*, *FAM117A*, *FZD2*, *GALNT12*, *GALNT3*, *GJB3*, *KDM6A*, *KYNU*, *PCNA*, *PFKP*, *PLEK2*, *RASGRP2*, *SERPIND1*, *SGSH*, *TLE1*, *TMEM38B*, *TMEM57*, *TRIM45*, *USP47*, *and VWA1*, at the threshold of *p*-value < 0.1 for MMPC algorithm. Finally, we built a multivariable Cox regression model on the 25 genes for overall survival prediction (Table 2). Based on the multivariable Cox regression model, risk score for each patient in the training set was calculated, and the 1,308 patients were classified into high- and low-risk groups. Kaplan–Meier curves showed that patients in the high-risk group had significantly shorter overall survival than those in the low-risk group (log-rank test *P* < 0.0001) (Fig. 3A).

**Table 2:** The estimation and hypothesis testing for the parameters of the gene signatures in multivariate Cox model.

Gene	coef	exp(coef)	se(coef)	z	Pr(>—z—)	Signif. codes
ABAT	−0.14	0.87	0.04	−3.50	4.65E−04	***
BCAR3	0.08	1.08	0.04	1.98	4.83E−02	*
CTSF	−0.06	0.94	0.04	−1.5	1.34E−01	
DEAF1	−0.09	0.92	0.04	−2.21	2.70E−02	*
ENC1	−0.12	0.89	0.04	−3.08	2.05E−03	**
ETV5	−0.09	0.92	0.04	−2.15	3.14E−02	*
FAM117A	0.08	1.08	0.04	2.03	4.29E−02	*
FZD2	−0.12	0.89	0.04	−3.09	2.02E−03	**
GALNT12	0.19	1.21	0.04	4.80	1.58E−06	***
GALNT3	0.02	1.02	0.04	0.47	6.37E−01	
GJB3	0.04	1.04	0.04	0.95	3.43E−01	
KDM6A	−0.14	0.87	0.04	−3.52	4.39E−04	***
KYNU	0.07	1.07	0.04	1.71	8.91E−02	
PCNA	0.06	1.07	0.04	1.60	1.09E−01	
PFKP	0.05	1.05	0.04	1.19	2.33E−01	
PLEK2	0.10	1.11	0.04	2.51	1.22E−02	*
RASGRP2	−0.05	0.95	0.04	−1.22	2.24E−01	
SERPIND1	−0.08	0.93	0.04	−1.90	5.79E−02	
SGSH	−0.06	0.94	0.04	−1.56	1.19E−01	
TLE1	0.05	1.05	0.04	1.33	1.84E−01	
TMEM38B	0.07	1.07	0.04	1.63	1.04E−01	
TMEM57	−0.08	0.92	0.04	−2.10	3.60E−02	*
TRIM45	0.16	1.17	0.04	3.97	7.32E−05	***
USP47	−0.07	0.93	0.04	−1.81	7.10E−02	
VWA1	−0.06	0.94	0.04	−1.51	1.30E−01	

In addition, we also investigated the performance of our stratification in specific stage, gender, and age group of LUAD in the training set. As no samples were stratified into TNM stage IV group in the training set, we only focused on the performance of the model in another three stages (I, II, and III). The overall survival difference between high- and low-risk groups in training set was observed in TNM stages I, II, and III, male/female, and old/young groups (Figs. 3B–3H, log-rank test, *P* < 0.005), in accordance with the performance in all samples. These results indicated that our stratification in training set was independent on TNM stages, gender, and age.

### Evaluation of the gene expression signature-based prognostic model in the validation sets

**Figure 4:**
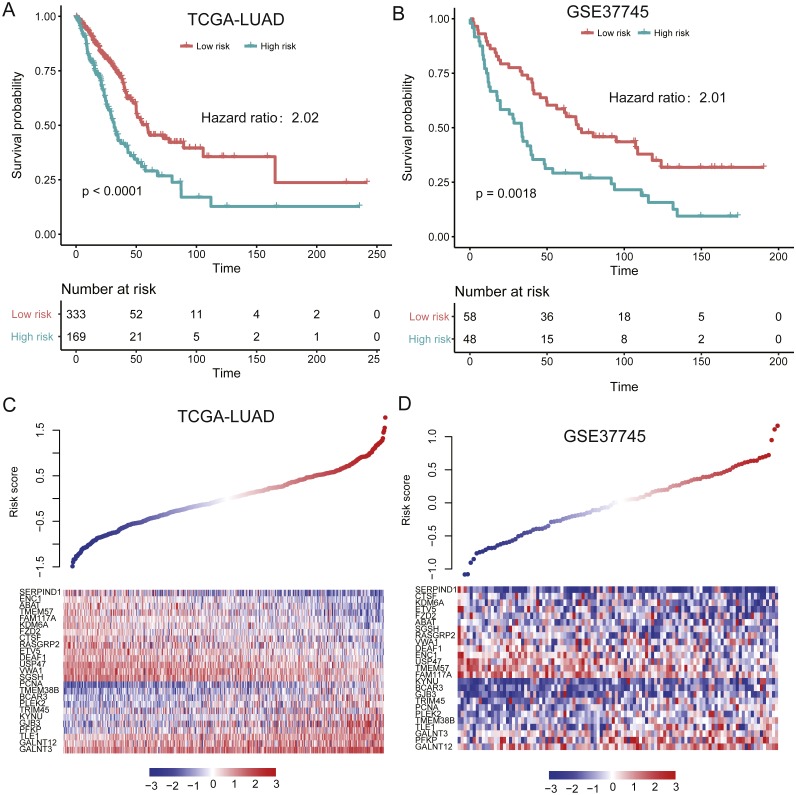
Performance of the prognostic model in two validation sets (TCGA and GSE37745). (A and B) illustrate the significant difference of the overall survival between the high- and low-risk groups. The signatures of 25 genes showed differentially expressed patterns in the two validation sets (C and D).

To evaluate the performance of the prognostic model in independent datasets, we collected two LUAD gene expression datasets, TCGA-LUAD (The Cancer Genome Atlas-lung adenocarcinoma, *n* = 502) (Cancer Genome Atlas Research N, 2014) and GSE37745 (*n* = 106) (Botling et al., 2013). The risk scores for the patients from validation sets based on the prognostic model were calculated. By using the same model and criteria, patients in the validation sets were classified into high-risk and low-risk groups. Similar with that in training set, the overall survival of the patients in high-risk group was significantly worse than that of low-risk group patients in the two validation sets (*P* < 0.001) (Figs. 4A–4B). Notably, the stratification still showed significant predictive ability in overall survival by adjusting the cofactors including age, gender, smoking status, tumor stage in TCGA cohort (*P* < 0.0001, Table 3). The distribution of the risk score, overall survival status along with the corresponding expression profiles of the 25 prognostic genes from two validation sets were showed in Figs. 4C–4D, which were ranked according to the risk score value. The 25 prognostic genes were significantly differentially expressed between the two risk groups (*P* < 0.05). The results indicated that the 25-gene signature based prognostic model showed high and robust performance in both training and the two validation sets.

### Evaluating the performance of gene expression signature-based prognostic model within TNM stages, gender, and age groups

**Figure 5:**
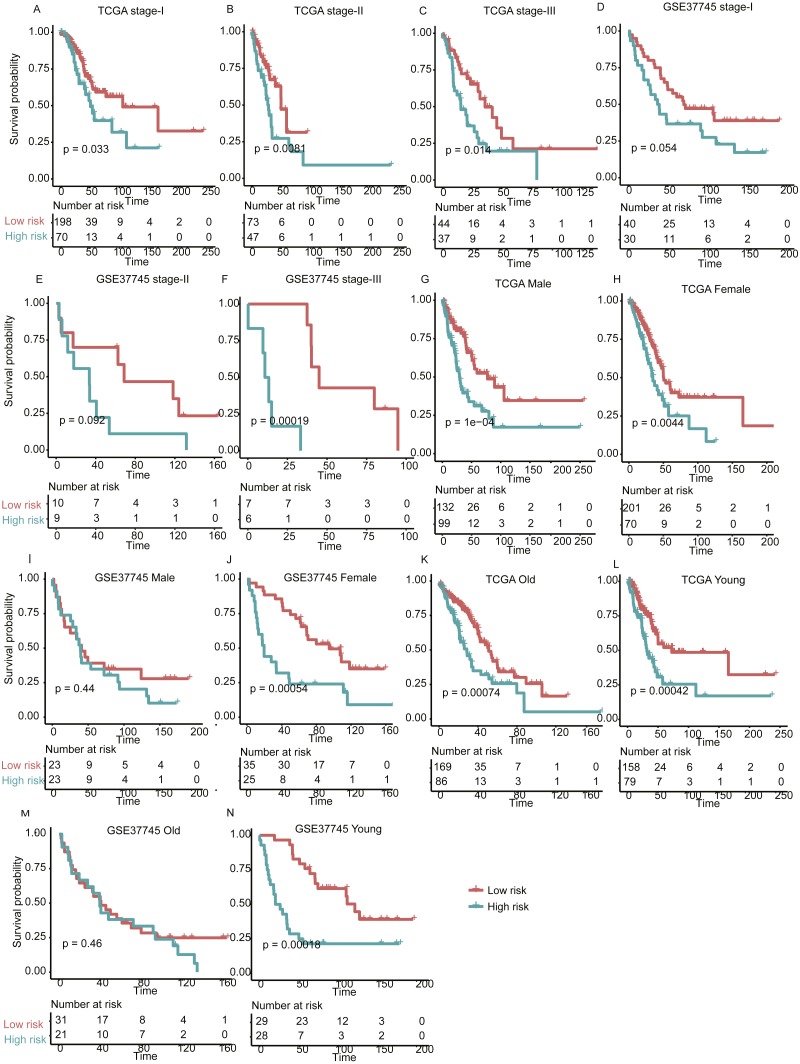
The performance of the prognostic model within TNM stages, age and gender group in the validation set. Overall survival differences between high- and low-risk groups are observed within specific TNM stage (A–F), gender (G–J), and age group (K–N).

With high performance of the gene expression signature-based prognostic model in all LUAD patients from both training and validation sets, it was also necessary to investigate its performance in specific stage, gender, and age group of LUAD. As no samples were stratified into TNM stage IV group in the training set, we only focused on the performance of the model in another three stages (I, II, and III). For validation of the prognostic prediction value within TNM stages, gender, and age groups, Cox regression coefficients and dichotomization cut-off threshold generated from the training set were directly applied to the two validation sets. Similarly, significant overall survival difference was observed between high- and low-risk groups with each TNM stage, male/female, and old/young age groups in both of the validation datasets (Fig. 5, *P* < 0.05), except samples in male and old group of GSE37745, which may be resulted from its small sample size. These findings further validate the robustness of the gene expression-based signatures in predicting survival in lung adenocarcinoma.

**Table 3:** The association adjusted by cofactors including age, gender, smoking status, and TNM stage between the stratification and the overall survival in TCGA-LUAD cohort.

	coef	exp(coef)	se(coef)	z	Pr(>—z—)	Signif.
Stratitification (HighRisk)	9.65E−01	2.63E+00	2.46E−01	3.922	8.79E−05	***
Age	1.83E−02	1.02E+00	1.21E−02	1.517	0.1292	
Gender (Male)	8.35E−02	1.09E+00	2.41E−01	0.346	0.72947	
Smoking (Yes)	−5.52E−01	5.76E−01	3.25E−01	−1.699	0.08939	
Stage (II)	8.11E−01	2.25E+00	2.96E−01	2.738	0.00618	**
Stage (III)	1.16E+00	3.20E+00	2.91E−01	3.997	6.41E−05	***

### Comparing signatures of 25 genes with known prognostic signatures in predicting LUAD prognosis

**Figure 6:**
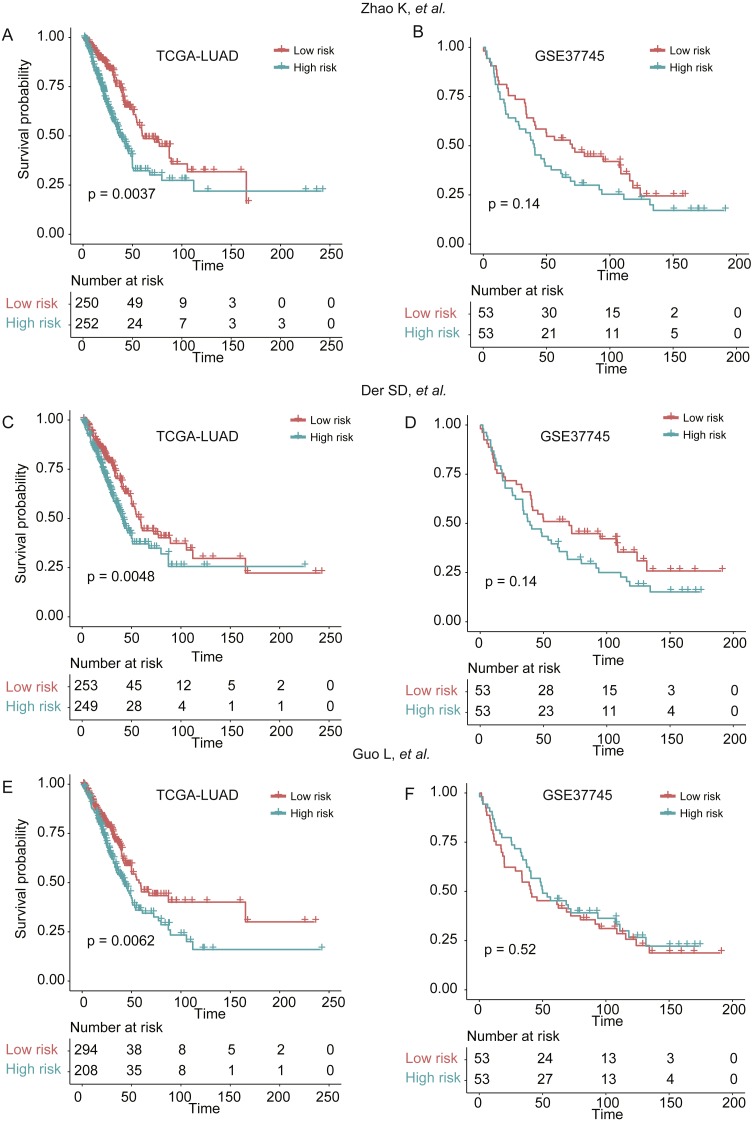
The performance of three prognostic gene sets in the two validation sets. The performance of the cox models built by three prognostic gene sets by Zhao, Li & Tian (2018), Der et al. (2014) and Guo et al. (2006) were visualized by Kaplan–Meier curves in (A and B), (C and D), and (E and F), respectively.

To demonstrate the robustness of the signatures of 25 genes in predicting LUAD prognosis, we built three more Cox models based on three signature gene sets found by previous studies (Der et al., 2014; Guo et al., 2006; Zhao, Li & Tian, 2018), which were selected from single dataset, and predicted the stratification of the two validation sets. We found that the three models showed worse ability in predicting the prognosis of patients in GSE37745 (Figs. 6B, 6D, and 6F), as compared with our signatures of 25 genes based Cox model (Fig. 4B), which may be caused by small sample size (*n* = 106). Although they had improved performance in TCGA-LUAD cohort (*n* = 502) (Figs. 6A, 6C and 6E), the significance levels of the three models were still worse than our model (Fig. 4A). These results suggested that the signatures of 25 genes were more robust than those selected by only one dataset.

## Discussion

The prognostic models for LUAD has been widely studied in the context of metastasis-free, organ-specific metastasis-free, and overall survival (Chen et al., 2018; Li et al., 2017; Park et al., 2012; Shukla et al., 2017). Despite extensive researches about the combinations of gene signatures selected for prognosis prediction, the lack of robust gene signatures for LUAD overall survival prediction is still not thoroughly solved. Meanwhile, the widespread use of high-throughput technologies produced a series of lung cancer gene expression datasets, which allowed us to integrate multiple datasets to comprehensively identify prognostic genes.

The present study aims to uncover a set of robust prognostic gene signatures and critical pathways. The ten LUAD gene expression datasets had long-term follow-up, which was more beneficial for us to carry out this research. To our knowledge, this is the first study that collects more than 1,300 samples for identification of prognostic signature and construction of prognostic model. The meta-analysis-based Cox regression analysis found 42 prognostic genes associated with overall survival, 25 of which were selected as predictors of multivariable Cox regression model by MMPC algorithm. GSEA identified Aurora-A pathway, Aurora-B pathway, and FOXM1 transcription factor network as prognostic pathways in LUAD. Moreover, the three prognostic pathways were also the biological processes of G2-M transition. It is well established that dysregulation of cell cycle checkpoints was a hallmark of cancer (Kastan & Bartek, 2004; Lam et al., 2004), suggesting that hyperactive G2-M transition in cell cycle was an indicator of poor prognosis in LUAD.

To examine the robustness of the prognostic model, we also calculated the risk scores for the patients from two validation sets. The further analysis suggested that overall survival differences were observed not only in all LUAD patients, but also in those with a specific stage, gender, and age group. Moreover, we also compared our signatures of 25 genes with those reported by three previous studies, and found that the significance levels of the three sets of signatures were still worse than our signatures of 25 genes (Fig. 6). In addition, the multivariable Cox model also highlights four highly predictive genes (*p*-value < 0.001, *ABAT, GALNT12, KDM6A,* and *TRIM45*), which may be useful for further experimental validation. The comprehensive analysis demonstrated that the prognostic signatures and prognostic model were robust in overall survival prediction.

In this study, our analysis demonstrated that large scale gene expression datasets could identify a set of robust gene signatures for overall survival prediction. Moreover, we also validated their predictive value in two independent datasets. This study indicates that meta-analysis-based prognostic feature selection might be an ideal strategy for the identification of prognostic gene signatures and construction of prognostic models.

## Conclusions

In summary, the prognostic gene signatures selected by meta-analysis-based Cox regression model and MMPC algorithm was more robust that those selected by single dataset. It is suggested that prognostic models based on these gene signatures could efficiently predict overall survival of LUAD patients.

##  Supplemental Information

10.7717/peerj.6980/supp-1 Supplemental Information 1 Code for the main data analysis

## Additional Information and Declarations

### Data Availability

The following information was supplied regarding data availability: The code is available in the Supplementary File.
